# The Role of Morcellation in En Bloc Resection of Large Bladder Tumors

**DOI:** 10.3390/diagnostics15060716

**Published:** 2025-03-13

**Authors:** Nadav Dekel, Ekaterina Laukhtina, Andrey Morozov, Eva Compérat, Eddie Fridman, Shay Golan, Jeremy Yuen-Chun Teoh, Yossef Molchanov, Maxim Yakimov, Thomas R. W. Herrmann, Dmitry Pushkar, Jesús Moreno Sierra, Juan Gómez Rivas, Shahrokh F. Shariat, Dmitry Enikeev

**Affiliations:** 1 Department of Urology, Rabin Medical Center, Petah Tikva 4941492, Israel; nadavdekel@gmail.com (N.D.); shaygo1@gmail.com (S.G.); 2 Faculty of Medicine, Tel Aviv University, Tel Aviv 69978, Israel; eddie.fridman@sheba.health.gov.il; 3 Institute for Urology and Reproductive Health, Sechenov University, Moscow 119991, Russia; katyalaukhtina@gmail.com (E.L.); andrei.o.morozov@gmail.com (A.M.); sfshariat@gmail.com (S.F.S.); 4 Department of Urology, Comprehensive Cancer Center, Medical University of Vienna, 1090 Vienna, Austria; 5 Department of Pathology, Hôpital Tenon, Sorbonne Université, 75006 Paris, France; evacomperat@gmail.com; 6 Department of Diagnostic Pathology, Sheba Medical Center, Ramat Gan 52621, Israel; yossef.molchanov@sheba.health.gov.il; 7 S.H. Ho Urology Centre, Department of Surgery, The Chinese University of Hong Kong, Hong Kong 999077, China; jeremyteoh@surgery.cuhk.edu.hk; 8 Pathology Department, Rabin Medical Center, Petah Tikva 4941492, Israel; dianaevening13@gmail.com; 9 Department of Urology, Spital Thurgau AG, Kantonspital Frauenfeld, Western Cape, 8596 Frauenfeld, Switzerland; thomas.herrmann@stgag.ch; 10 Division of Urology, Department of Surgical Sciences, Stellenbosch University, Stellenbosch 7602, South Africa; 11 Hannover Medical School, 30625 Hannover, Germany; 12 Department of Urology, Moscow State University of Medicine and Dentistry (MSMU), Moscow 127473, Russia; pushkardm@mail.ru; 13 Department of Urology, Hospital Clínico San Carlos, 111821 Madrid, Spain; dr_jmoreno@hotmail.com (J.M.S.); juangomezr@gmail.com (J.G.R.); 14 Hourani Center for Applied Scientific Research, Al-Ahliyya Amman University, Amman 19111, Jordan; 15 Department of Urology, University of Texas Southwestern Medical Center, Dallas, TX 75390, USA; 16 Department of Urology, Weill Cornell Medical College, New York, NY 10065, USA

**Keywords:** NMIBC, TURBT, ERBT, morcellation

## Abstract

**Background/Objectives**: Conventional transurethral resection of bladder tumor (TURBT) for non-muscle invasive bladder cancer (NMIBC) is usually performed in a piecemeal manner, leading to difficulties in accurate pathological assessment. En bloc resection of bladder tumor (ERBT) has been developed to address these limitations, offering improved specimen quality. So far, ERBT has been restricted to small bladder tumors due to difficulties in en bloc extraction of large ones (>3 cm). Recently, the morcellation technique has been proposed to facilitate the removal of large bladder tumors during ERBT. This narrative review aims to evaluate the feasibility of ERBT with subsequent morcellation for large bladder tumors, focusing on its role in tumor extraction and its impact on pathological assessment. **Methods**: A comprehensive literature search was conducted across multiple databases to identify studies evaluating the use of morcellation in ERBT for large bladder tumors. Inclusion criteria comprised studies reporting recurrence rates, detrusor muscle (DM) presence in pathological specimens, and perioperative complications. Additionally, we offered uropathologists a questionnaire to gather their perspectives on the use of morcellation following ERBT, focusing on its impact on pathological assessment, margin evaluation, and staging accuracy. **Results**: While there is limited evidence on the use of morcellation in ERBT for tumors larger than 3 cm and its impact on oncologic outcomes, morcellation has shown potential in facilitating the retrieval of large tumor specimens, ensuring clear resection margins and accurate staging. However, the learning curve for morcellation techniques and the need for specialized equipment may limit widespread adoption. **Conclusions**: Morcellation in ERBT for large bladder tumors represents a promising advancement in the management of these challenging cases, offering adequate pathological assessment and oncologic outcomes. Pathologists’ reviews of morcellated specimens will likely further validate the technique. Continued research and technological innovations are necessary to optimize its implementation in clinical practice.

## 1. Introduction

Bladder cancer is a prevalent urological malignancy [1]; it poses an increasing challenge for patients, healthcare providers, and society at large. Globally, it ranks 7th among the most common malignancies in men and 11th in both sexes combined [1]. Non-muscle invasive bladder cancer (NMIBC) accounts for approximately 75% of all bladder cancer diagnoses. Despite advances in imaging, pathology remains the most reliable and accurate staging tool. Given the highly variable clinical course of NMIBC, precise histopathological assessment is essential for developing the best management plan tailored to each patient [2].

Conventional transurethral resection of bladder tumor (TURBT) provides detailed information on the extent of the tumor, although it may be difficult for pathologists to orient the tissue correctly and thus assess the surgical margin and depth of invasion. However, performing high-quality TURBT is challenging, as evidenced by unsatisfactory outcomes such as residual tumor rates of 46–56% and varying detrusor muscle (DM) presence in specimens, ranging from 30–100% [3,4]. Moreover, diathermy used in the resection process can cause cautery and crush artifacts, which alter the microscopic appearance of the tissue, potentially leading to misinterpretations and loss of critical information [3,4]. En bloc resection of bladder tumor (ERBT), which can be performed using electrocautery or lasers, has been invented to improve specimen quality with reported DM presence in 96–100% of cases [5]. So far, ERBT has been restricted to small bladder tumors due to difficulties in en bloc extraction of large bladder tumors (>3 cm). To address this, several options have been suggested, with tumor morcellation being one of them.

This narrative review aims to evaluate the role of morcellation in ERBT for large bladder tumors, focusing on its effectiveness in achieving an accurate pathological assessment.

## 2. Materials and Methods

A comprehensive literature search was conducted across multiple databases to identify studies evaluating the use of morcellation in ERBT for large bladder tumors. The following words were used in the search query: bladder tumor or lesion, NMIBC, ERBT, TURBT, transurethral resection, en bloc, morcellation, large tumor, etc. Inclusion criteria comprised studies reporting recurrence rates, detrusor muscle (DM) presence in pathological specimens, and perioperative complications. Additionally, we offered uropathologists a questionnaire to gather their perspectives on the use of morcellation following ERBT, focusing on its impact on pathological assessment, margin evaluation, and staging accuracy.

## 3. Results

### 3.1. ERBT Versus Conventional TURBT

Standard TURBT is performed in a piecemeal manner, which sometimes makes it impossible to assess the resection margins accurately [6]. The presence of the DM in the pathology specimen is one of the key quality indicators for TURBT. The DM helps ensure staging accuracy, detect residual tumors during subsequent TURBTs, and predict early recurrence [3]. Residual tumors are rather common; a study by Cumberbatch et al. found residual disease in 17–67% of Ta tumors and 20–71% of T1 tumors, while DM presence ranged from 30% to 100% in different studies [5].

Another quality indicator for TURBT is complete tumor resection. Even though achieving “complete resection” heavily relies on the surgeon’s experience, TURBT often fails to completely remove the tumor [6]. Between 30% and 60% of T1 tumors initially treated with TURBT are discovered to be muscle-invasive at the time of radical cystectomy [4]. Performing a second-look TURBT enhances diagnostic accuracy and helps to remove residual cancer. The rate of tumor upstaging varies, but approximately 5–10% of patients initially diagnosed with T1 tumors are found to have muscle-invasive cancer upon second-look TURBT [3,5,7].

Among other considerations, it should be noted that TURBT carries significant perioperative risks, including the obturator nerve reflex, bladder perforation, and bleeding, which add to the overall risk [6,8].

ERBT addresses many disadvantages of TURBT by removing the tumor in one piece, thereby reducing the risk of tumor cell reimplantation and ensuring complete tumor resection. En bloc resection offers greater precision and control, potentially reducing complications such as bladder perforation [4,9]. This technique adheres to fundamental principles of cancer surgery, ensuring thorough and effective tumor removal with negative resection margins achieved in 94–99% of cases [4,9]. ERBT significantly increases the presence of the DM in pathological specimens, regardless of the energy source used [7].

In terms of oncologic outcomes, a randomized controlled trial (RCT) by Teoh et al. demonstrated a statistically significant lower 1-year recurrence rate in the ERBT group (29%) compared to the standard TURBT group (38%) among NMIBC patients with tumors smaller than 3 cm [10]. This contrasts with the results of two other RCTs that did not show a significant difference in recurrence rates between the two surgical methods [11,12] but did show higher rates of DM presence, less residual tumor on repeat TURBT, and a better safety profile in favor of ERBT [2]. In a prospective study of 129 NMIBC patients, Enikeev et al. demonstrated that the recurrence rates at 3 and 6 months post-surgery were lower with ERBT compared to conventional TURBT [13].

Therefore, ERBT seems to be a viable alternative of the surgical management of NMIBC, providing a more accurate pathological assessment compared to traditional TURBT, although its impact on oncologic outcomes remains controversial.

### 3.2. ERBT Techniques

At the beginning of the ERBT procedure, a circumferential margin is pileated and incised, maintaining a distance of at least 5 mm from the visible edge of the tumor [9,14]. It is essential to cut down to the detrusor muscle layer, which causes the tumor to contract toward the center as the mucosa and submucosa are released. The tumor base is managed with both forward and backward dissection using a combination of sharp and blunt techniques. The tumor is usually then removed entirely in one piece, sometimes with the assistance of grasping forceps or an endo-bag, or occasionally in multiple segments in a modified ERBT approach [14].

#### 3.2.1. Energy Sources

Various energy sources for ERBT have been described, such as the following:

1. Electrocautery. This method utilizes either mono- or bipolar energy to perform tumor resection.
2. Laser-based ERBT (L-ERBT). Several laser systems can be employed for this technique, including holmium:YAG (Ho:YAG), GreenLight, diode, thulium:YAG (Tm:YAG), and thulium fiber laser (TFL).
3. Hydrodissection. This approach uses a high-pressure saline stream to dissect the tissue beneath the tumor lesion, followed by an incision made with monopolar energy.

Each technique offers unique advantages and considerations for effective tumor resection [14]. Both the electrocautery and laser techniques are effective at achieving accurate staging. The presence of the DM in ERBT specimens varies, with rates of 87–98% for laser, 40–100% for monopolar, and 51–100% for bipolar electrocautery [15].

Yongliang Lu et al. reported a recurrence rate of 22.7% during a median follow-up of 80 months after ERBT using a 2 µm thulium laser, highlighting its effective curative potential [16,17,18,19]. This technique offers DM detection rates of up to 90% and identifies more instances of T1 disease compared to traditional TURBT [5,20]. The resection time of laser ERBT is comparable to conventional TURBT, but it offers advantages such as a shorter catheterization duration, absence of the obturator reflex, and improved accuracy in local staging. This is achieved through a better sampling of the DM, with a 98% success rate after the laser ERBT procedure compared to 62% after conventional TURBT [21,22].

#### 3.2.2. Tumor Characteristics

The number of bladder tumors does not significantly limit the performance of ERBT. Most studies consider four tumors as the threshold for ERBT, though it may require more time and effort.

In terms of tumor size, most studies set a 3 cm cut-off for tumor size, as extracting larger specimens in one piece can be challenging. Nevertheless, ERBT ensures complete local resection even if the specimen cannot be retrieved intact [9].

It is generally feasible to perform ERBT on tumors located in all areas of the bladder; it is also safe to administer a single dose of immediate intravesical chemotherapy, perform a second-look TURBT, and provide adjuvant intravesical Bacillus Calmette–Guérin (BCG) therapy after ERBT [9].

### 3.3. Tumor Extraction During ERBT

A significant challenge for ERBT is handling larger tumors. As the size of the lesion increases, it becomes more difficult to clearly visualize the tumor margins and maintain an accurate dissection plane throughout the procedure. Moreover, the removal of large tumors is complicated by the fact that they may not fit through the endoscope channel, necessitating additional techniques or equipment to extract the tumor effectively. These factors make ERBT more complex when dealing with large bladder tumors. When grouped by tumor size, ERBT achieved a technical success rate of 84.3% for tumors 3 cm or smaller, while for those exceeding 3 cm, the success rate dropped to 29.6% [23].

One of the technical modifications for retrieving specimens up to 4.5 cm was introduced by Angelo et al. [24]. After making an incision around the lesion with a Collins loop, the muscular fibers are sectioned from the periphery toward the center of the lesion base. Once the lesion is detached, 5 mm laparoscopic forceps are inserted through the working channel to grasp the tumor. The resectoscope is then carefully withdrawn from the bladder through the urethra. This technique helps maintain the integrity of large lesions during extraction [24].

Bags or endobags can also offer an effective solution for extracting large tumors during ERBT. They allow safe removal of tumors by enclosing them after dissection. The endobag technique separates the tumor from the bladder mucosa, preventing contact and potential contamination. Despite the tumor size, its malleable nature allows it to change shape within the bag, facilitating passage through the endoscope channel without causing structural damage or spreading tumor cells. However, this method may be more complex when dealing with particularly solid lesions [25].

### 3.4. Morcellation: Technique and Application

The electromechanical, or power morcellator, was introduced in 1993, with the morcellation technique for prostatic tissue retrieval following transurethral enucleation first described in 1998 [26]. Morcellation involves mechanical disintegration of large tissue masses into smaller fragments that can be easily removed through the resectoscope.

Morcellation is widely used across various fields in urology, including prostate enucleation, ERBT, and even following nephrectomy. Despite the fragmented appearance of morcellated specimens, accurate histopathological and staging information can still be obtained. Pathologists can reliably determine the diagnosis, grade, stage, percentage of tumor involvement, and presence or absence of vascular invasion using morcellated tissue [27,28].

There are three widely used stand-alone morcellation devices, such as the DrillCut (Karl Storz, Tuttlingen, Germany), which features a rotating toothed blade; the Piranha (Richard Wolf, Knittlingen, Germany), with an oscillating toothed blade; and the VersaCut (Lumenis, Santa Clara, CA, USA), which employs a nontoothed guillotine blade. For the Piranha device, studies report a weighted mean efficiency of 5.29 g/min (range 2.7–20 g/min). At 1500 rpm, disposable blades significantly enhance morcellation efficiency compared to reusable blades, as the device suctions and captures tissue fragments effectively [29].

Urologists choose morcellation devices based on factors like safety, speed, ease of use, reusability, and cost-effectiveness. Studies indicate that the Piranha morcellator provides superior efficiency, has a relatively simple learning curve, and maintains a solid safety profile. This leads to reduced operating room times and lower overall costs compared to other available devices [30,31].

It is important to note that during the morcellation procedure, complications such as bladder wall injury, morcellator malfunction, and poor visualization can occur. Additionally, the use of different instruments (e.g., a monopolar loop, cystoscopic forceps, or a grasper) may be necessary to retrieve small tissue fragments [26].

Recently, the morcellation technique, widely used in different surgical fields, has been adapted for use in ERBT to facilitate the removal of large bladder tumors, including those exceeding 5 cm in size [32].

### 3.5. Advantages of Morcellation

There are several advantages of ERBT with morcellation for large tumors. The first is reduced tissue fragmentation, as morcellation ensures that large tumors are broken down into manageable pieces. The second is enhanced retrieval: the suction of the morcellator allows for complete removal of tumor tissue with less scattering of tumor cells. The third is time efficiency: while morcellation may be slower than simply removing the tumor through the endoscope channel, it is typically faster for tumors that would otherwise require removal using forceps, resecting the “en-blocked” tumor into small fragments for endoscopic removal, or a retrieval bag, thereby reducing the overall operative time in such cases.

### 3.6. Pathological Aspects of ERBT with Morcellation

#### 3.6.1. Histopathological Evaluation

Tissue samples obtained from conventional TURBT are sometimes of poor quality, making it difficult for pathologists to interpret the results accurately. Issues such as inadequate specimen collection and thermal artifacts prevent the accurate staging and assessment of complete cancer removal [33]. ERBT, however, causes less thermal damage and preserves tissue integrity. Moreover, it allows for lesser penetration depth when using a laser. In a study by Enikeev et al., the DM was found in 91% of Tm-fiber ERBT cases compared to only 58% in conventional TURBT cases [13]. In large tumors, according to a study conducted by Petov et al., which compared ERBT to conventional TURBT in patients with bladder tumors larger than 3 cm, the DM was found in 92.8% of cases in the ERBT group, in contrast to only 70.5% in the TURBT group [34].

Furthermore, according to a recent meta-analysis, ERBT enhances the detection rate of the muscularis mucosae in specimens [2]. This improvement allows for more precise T1 substaging, leading to better predictions of recurrence and progression. Furthermore, ERBT has been shown to result in a lower residual tumor rate at the second-look TURBT compared to conventional TURBT, suggesting a more complete initial tumor resection. Additionally, patients with negative horizontal and vertical margins (pT1) exhibited no residual tumors at the second-look TURBT [2,35].

Interestingly, according to a study by Guven et al. involving 68 pathologists from 23 countries, 73% of pathologists expressed a view that en bloc tissue samples improve the accuracy of final pathology staging. Despite this, only 38% reported occasionally receiving tumors larger than 3 cm after the en bloc technique [36].

Morcellation of removed benign prostatic hyperplasia (BPH) tissue is standard practice following most endoscopic prostate enucleation procedures, and its use provides valuable insights into ERBT. Studies comparing histological evaluations between holmium laser enucleation of the prostate (HoLEP) and transurethral resection of the prostate (TURP) demonstrate that morcellation yields sufficient tissue for accurate pathological analysis without compromising the pathologist’s ability to detect prostate cancer. The overall tissue structure is preserved, with most of the damage caused by the laser rather than the morcellation process, leading to outcomes comparable to TURP [37]. Misrai et al. compared prostate cancer detection between GreenLEP followed by morcellation and open simple prostatectomy; no significant differences in prostate cancer detection, staging, or Gleason scores were observed [38]. This indicates that morcellation does not impede adequate pathological evaluation and might be a reliable method for preserving tissue integrity and ensuring diagnostic accuracy during the ERBT procedure.

#### 3.6.2. Perspectives from the Pathologists on the Impact of Morcellation in ERBT

To gain insight into the pathological assessment of bladder cancer from ERBT specimens following morcellation, we asked for the opinion of three dedicated uropathologists (Table 1). These pathologists have expertise in evaluating tissues from various urological surgeries, including post-morcellation procedures such as HoLEP and ERBT. The survey aimed to understand how morcellation impacts their ability to assess critical factors in bladder cancer staging.

This survey highlights that while morcellation presents some challenges, its overall impact on pathological assessment is minimal. The ability to assess depth of invasion, lymphovascular invasion, and DM presence remains intact, making morcellation a useful and reliable technique in the context of ERBT for large tumors.

### 3.7. Impact on Oncologic Outcomes

For patients with NMIBC and tumors up to 3 cm, ERBT has been shown to yield a higher incidence of DM in pathological specimens compared to TURBT (81% vs. 71%). Additionally, the disease persistence rate at second-look TURBT is 6% lower with ERBT than with TURBT. However, no significant difference in recurrence rates has been found between ERBT and TURBT in an RCT by D’Andrea [11]. Contrary to these findings, an RCT by Teoh et al. [11] observed a significant reduction in the 1-year recurrence rate with ERBT compared to standard TURBT (29% vs. 38%) in NMIBC patients with tumors sized smaller than 3 cm. Recently, Gallioli et al. [12] reported results of a noninferiority RCT that compared ERBT with conventional TURBT in 300 NMIBC patients. Their findings indicated that the presence of DM was similar between the ERBT and TURBT groups (94% vs. 95%). The study also noted recurrence rates of 13% for the ERBT group and 18% for the TURBT group after a 15-month follow-up period.

A key consideration in the management of high-grade (HG) disease is the role of second-look TURBT. The primary rationale for this approach stems from reports indicating that up to 30% of T1 tumors resected via TURBT may actually harbor muscle-invasive disease [39]. However, most of the existing evidence is derived from studies on standard TURBT. Residual tumors after conventional TURBT have been reported in up to 75% of patients with Ta and T1 disease. Upstaging from Ta to T1 or from T1 to T2 disease during second-look TURBT has been reported in 28% of cases with initial T1 tumors and 9.5% of those with initial TaHG tumors. In contrast, second-look TURBT following ERBT showed a residual tumor rate of 5.9% and no cases of upstaging. Second-look TURBT after ERBT reveals relatively low rates of residual disease and upstaging and does not appear to impact recurrence or progression rates [40]. A retrospective study by Levy et al. involving 75 patients who underwent a second-look TURBT following ERBT for T1HG urothelial carcinoma found no upstaging to T2 disease at the primary T1 tumor site. However, a considerable rate of residual disease (29%) was detected in other areas of the bladder [39]. The likelihood of residual tumor was higher in patients with multiple tumors and those with CIS. The authors suggested that in patients without these risk factors (multiple tumors and/or CIS), the probability of residual disease is very low, making them potential candidates for replacing routine second-look TURBT with non-invasive monitoring techniques, such as outpatient cystoscopy, cytology, and/or biomarkers [39].

The use of morcellation in ERBT presents several theoretical advantages that could influence tumor recurrence and progression. By accurately disintegrating and removing large tumors, morcellation is thought to reduce the risk of tumor cell scattering, a key factor in the recurrence of bladder cancer, as mentioned earlier in this review. The assumption is that by minimizing the potential spread of cancerous cells during extraction, morcellation may contribute to lower recurrence rates in both the bladder and the urethra. However, these potential benefits are largely based on theoretical assumptions. The actual impact of morcellation on oncologic outcomes in NMIBC patients remains to be conclusively determined.

While existing evidence underscores the possible advantages of ERBT, it is important to note that the evidence supporting the use of morcellation in this context is limited. There is a significant lack of oncological data specifically addressing the outcomes associated with morcellation, and its role in NMIBC management remains inadequately studied. Further research is necessary to establish the oncological safety and efficacy of the morcellation approach within the ERBT procedure.

## 4. Discussion

### 4.1. Complications and Challenges

Building on insights from studies in the field of transurethral enucleation of the prostate, the use of morcellation in ERBT surgery presents certain challenges. The effectiveness of tissue removal during morcellation depends on several factors, including tissue consistency, suction power, and the specific design of the morcellator blade [41]. While the procedure is carried out with specialized equipment and under careful endoscopic view using a nephroscope to minimize the risk of bladder injury, potential complications can still arise [29]. These may include damage to the bladder mucosa, perforation, or, on occasion, delays in the morcellation process due to technical issues with the device. Additionally, even minor bleeding can impair visibility, making the procedure more complex and increasing the level of difficulty [41,42].

It is important to highlight that, during prostate enucleation, two water inflows are typically employed via the nephroscope to maintain bladder distension, counteracting the strong suction generated by the morcellator and thereby reducing the risk of injury [43]. However, this precaution is not utilized in ERBT, which elevates the potential for bladder wall injury. Therefore, finding a balance between the speed of morcellation and the necessary safety measures is essential.

### 4.2. Cost and Availability of Tools and Facilities

The data about cost-effectiveness of morcellation in endourology are significantly lacking. We identified only one study assessing the difference in morcellation cost in BPH surgery [30]. Remarkably, the authors noticed that a morcellator was chosen predominantly based on its safety, efficiency, and ease of use, while the cost and reusability were not so important. No such studies for the morcellation of bladder tumors were conducted.

As we mentioned previously, ERBT may be performed by electrocautery and thus theoretically does not require an additional generator or expensive tools compared with TURBT. However, the majority of urologists prefer laser energy due to its advantages. A laser is an expensive device, but we should keep in mind that most of the modern urological lasers are multi-purpose and effective for soft-tissue surgery (for bladder tumors and BPH) as well as for lithotripsy. We believe that ERBT with morcellation is primarily justified in a urological center where these three types of procedures are performed.

### 4.3. Learning Curve

Accurate tumor staging and complete removal of all visible tumors rely significantly on the surgeon’s expertise and confidence in performing a sufficiently wide and deep resection of the bladder tumor to obtain the DM. In this context, the ability to resect the DM varies between surgeons. Jesuraj et al. reported that for TURBT, the DM was present in 45.8% of resections performed by junior surgeons compared to 67.3% by senior surgeons [8,44]. Poletajew et al. suggested that TURBT exhibits a learning curve, with a minimum of 100 cases required for a resident in training to achieve satisfactory oncological and surgical outcomes [45].

When considering the learning curve for the morcellation procedure, according to Misrai et al., proficiency with the Piranha morcellator typically requires about 30 procedures, while achieving maximum efficiency takes around 60. Notably, the safety of the procedure improves with the experience of both the surgeon and the medical team [46]. Complications were primarily observed during the initial stages of the learning curve, with injury rates decreasing as practitioners gained more expertise [46]. Unfortunately, the data regarding learning curve are retrieved from the investigations on BPH tissue morcellation. The absence of a direct comparison between learning curves for TURBT vs. ERBT with morcellation is a limitation in current knowledge. However, bladder tumors are usually much softer compared with prostate tissue, and their morcellation does not cause such difficulties as “beach ball” morcellation in the case of prostate enucleation, requiring high experience. Pathologists also believe that en bloc specimens allow for quicker, more accurate, and cost-efficient diagnoses [36].

In summary, the use of morcellation in ERBT involves several challenges, including several factors such as tissue consistency, suction power, and morcellator design, which can increase the risk of complications like bladder mucosa damage or perforation. Minor bleeding can also hinder visibility, making the procedure more difficult. A key point is finding the right balance between morcellation speed and necessary safety measures. Proficiency in the procedure requires experience, with a learning curve of 30 to 60 cases to achieve optimal efficiency and safety. Additionally, pathologists face a steeper learning curve when assessing piecemeal specimens, though en bloc samples allow for quicker, more accurate diagnoses.

## 5. Conclusions

Morcellation during ERBT represents a promising advancement in the management of large bladder tumors, offering adequate pathological assessment and oncologic outcomes. Given that ERBT specimens frequently contain the detrusor muscle and achieve clear margins at a higher rate compared to conventional TURBT, it may reduce the need for additional procedures, such as second-look TURBT, even for large bladder tumors, and could also help lower treatment costs for patients with NMIBC. Continued research and technological innovation will be crucial in optimizing this technique and ensuring its safe and effective implementation in clinical practice.

## Figures and Tables

**Table 1:** Pathologists’ survey.

Survey Question	Pathologists’ Answer
Margin Assessment	Margins are impossible to assess with morcellation, but they are less important for staging. Depth of invasion and detrusor muscle presence can still be evaluated.
Effect on Lymphovascular Invasion (LVI) Detection	LVI detection is not significantly compromised by morcellation. Pathologists can still identify LVI with high accuracy.
Detrusor Muscle (DM) Evaluation	Morcellation does not impair the ability to assess detrusor muscle presence, which remains reliable for accurate staging.
Specimen Integrity and Quality	Morcellated specimens from ERBT are of higher quality due to laser use, which causes less tissue damage compared to electrocautery.
Risk of Artifacts and Misinterpretation	No significant artifacts or distortions are introduced by morcellation that would affect pathological interpretation.
Overall Opinion on Morcellation in ERBT	Morcellation in ERBT provides high-quality specimens, allowing accurate staging and DM evaluation. Pathologists find it beneficial despite challenges with margins.

## Data Availability

All of the data are provided within the manuscript.

## Abbreviations

The following abbreviations are used in this manuscript:

TURBT	transurethral resection of bladder tumor
NMIBC	non-muscle invasive bladder cancer
ERBT	en bloc resection of bladder tumor
DM	detrusor muscle
